# The Infectious Disease Physician

**DOI:** 10.1155/S1064744997000549

**Published:** 1997

**Authors:** Sebastian Faro


Infectious Diseases in Obstetrics and Gynecology 5:317 (1997)
© 1998 Wiley-Liss, Inc.

### The Infectious Disease Physician

The obstetrician-gynecologist who specializes in infectious diseases has a responsibility to patients, other physicians, residents, medical students, and industrial leaders. The infectious disease physician's clinical responsibilities cross all specialties with obstetrics and gynecology. We have spent a great deal of time writing, giving CME courses, special symposia and lectures, teaching residents and medical students, and performing research. The subspecialty of infectious diseases has come a long way since its conception.

Looking back over the years, the subspecialty has not provided guidelines for our fellow obstetricians-gynecologists. Unfortunately, not providing recommendations based on a consensus has created confusion with regard to diagnosis and treatment of infectious diseases for the obstetric and gynecologic patient. Therefore, it is time for the infectious disease specialists to come together and develop recommendations for the diagnosis, management, and treatment of infectious diseases in obstetrics and gynecology. Because of changes that have taken place in clinical research, it is time for the infectious disease specialists to take an active role in the development of research protocols sponsored by industry. This activity will not only benefit industry, but will have a positive effect on the health and well being of our patients.

The group of individuals who make up the infectious disease community should develop a network of researchers and a multi-center research association to work together on meaningful research projects. An approach such as this will produce an understanding of the pathophysiology of the infectious disease problems unique to women. The climate is right for this group of dedicated and learned individuals to approach the study of infectious diseases in an organized and well-directed manner to improve women's health through both research and teaching our colleagues the most efficient and cost-effective means to achieve these objectives.

*Sebastian Faro, MD, PhD*
*Editor-in-Chief*

